# The effect of strand bias in Illumina short-read sequencing data

**DOI:** 10.1186/1471-2164-13-666

**Published:** 2012-11-24

**Authors:** Yan Guo, Jiang Li, Chung-I Li, Jirong Long, David C Samuels, Yu Shyr

**Affiliations:** 1 Vanderbilt Ingram Cancer Center, Center for Quantitative Sciences, Nashville, TN, USA; 2 Vanderbilt Epidemiology Center, Vanderbilt University School of Medicine, Nashville, TN, USA; 3 Center for Human Genetics Research, Vanderbilt University, Nashville, TN, USA

**Keywords:** Next Generation Sequencing, Strand Bias, Illumina, Short Read, SNP quality control

## Abstract

**Background:**

When using Illumina high throughput short read data, sometimes the genotype inferred from the positive strand and negative strand are significantly different, with one homozygous and the other heterozygous. This phenomenon is known as strand bias. In this study, we used Illumina short-read sequencing data to evaluate the effect of strand bias on genotyping quality, and to explore the possible causes of strand bias.

**Result:**

We collected 22 breast cancer samples from 22 patients and sequenced their exome using the Illumina GAIIx machine. By comparing the consistency between the genotypes inferred from this sequencing data with the genotypes inferred from SNP chip data, we found that, when using sequencing data, SNPs with extreme strand bias did not have significantly lower consistency rates compared to SNPs with low or no strand bias. However, this result may be limited by the small subset of SNPs present in both the exome sequencing and the SNP chip data. We further compared the transition and transversion ratio and the number of novel non-synonymous SNPs between the SNPs with low or no strand bias and those with extreme strand bias, and found that SNPs with low or no strand bias have better overall quality. We also discovered that the strand bias occurs randomly at genomic positions across these samples, and observed no consistent pattern of strand bias location across samples. By comparing results from two different aligners, BWA and Bowtie, we found very consistent strand bias patterns. Thus strand bias is unlikely to be caused by alignment artifacts. We successfully replicated our results using two additional independent datasets with different capturing methods and Illumina sequencers.

**Conclusion:**

Extreme strand bias indicates a potential high false-positive rate for SNPs.

## Background

Over the last few years, high throughput sequencing technology has matured technically while becoming more affordable and is now the preferred approach for the discovery of novel mutations and differentially expressed genes. Even though the data produced by high throughput sequencing are richer and more informative than data generated from other traditional high throughput genomic technologies, the post analysis phase of next generation sequencing data presents numerous novel difficulties. A primary challenge associated with sequencing data analysis is the accurate detection of single nucleotide polymorphism (SNP)/mutation. Many issues can affect SNP/mutation detection accuracy. In this study, we focused on strand bias, one of the many issues concerned with SNP/mutation detection. Strand bias occurs when the genotype inferred from information presented by the forward strand and the reverse strand disagrees. For example, at a given position in the genome, the reads mapped to the forward strand support a heterozygous genotype, while the reads mapped to the reverse strand support a homozygous genotype (examples are given in Table 1). Due to the lack of transparency in the sequencing analysis pipeline and because most analysis tools will only report the final variant results, the issue of strand bias is not widely known. No standard analysis programs report nucleotide counts separately by the forward and reverse strands. However, strand bias actively concerns some researchers and developers of sequencing analysis and they have devoted some effort to measure strand bias. For example, the widely used Genome Analysis Tool Kit (GATK) [1], developed by the Broad Institute, calculates a strand bias score for each SNP identified. Another well-known sequencing analysis program, Samtools [2], also computes a strand bias score based on Fisher's exact test. The common practice has been to either ignore strand bias due to lack of knowledge or to use strand bias as a filter to reduce false-positive SNPs, thus reducing the overall call rate. However, to what extent strand bias may adversely affect genotyping quality in sequencing data has not been studied thoroughly. Thus, we designed this study to examine the effect of strand bias on genotyping quality and tried to identify the causes of strand bias.

**Table 1:** Strand bias examples from real data

**Chr**	**Pos**	**Depth**	**a**^**1**^	**b**^**2**^	**c**^**3**^	**d**^**4**^	**Forward Strand Genotype**	**Reverse Strand Genotype**
6	32975014	21	5	5	10	1	Heterzygous	Homozygous
1	81967962	38	20	11	7	0	Heterzygous	Homozygous
12	10215654	31	15	9	7	0	Heterzygous	Homozygous

^1^. Forward strand reference allele.

^2^. Forward strand non reference allele.

^3^. Reverse strand reference allele.

^4^. Reverse strand non reference allele.

Unbalanced strand mapping is a phenomenon when the number of reads mapped to forward and reverse strands are significantly different. In extreme cases, all reads are mapped to one strand, leaving the other strand completely uncovered. Unbalanced strand mapping is also considered to be a type of strand bias. Fundamentally, however, it is a different problem from strand bias in the calls. In a previous study [3], we have shown that unbalanced strand mapping is an artifact of the exome capturing mechanism and does not affect the quality of the genotyping. Thus, in the current study we focused only on strand bias related to the genotype call difference between the forward and reverse strands.

## Methods

### Data description and processing

We randomly selected whole exome sequencing data for 22 breast cancer patients recruited to the Shanghai Breast Cancer Study (SBCS). The SBCS is a large, population-based case–control study of women in urban Shanghai, the details of which have been previously described [4,5]. All patients had very early-onset (22–32 years old) breast cancer or early-onset (38–41 years old) plus a first-degree family history of breast cancer. Approval of the study was granted by the relevant institutional review boards in both China and the United States. Genomic DNA from buffy coat samples was extracted using QIAmp DNA kit (Qiagen, Valencia, CA) following the manufacturer’s protocol. All samples have been genotyped using the Affymetrix 6.0 array in a previous genome wide association study [4]. All patients in this study signed written informed consent. The approvals of the study were given by the institutional review board of Shanghai Cancer Institute and Vanderbilt University.

Sequencing was performed at the Genome Service Lab at Hudson Alpha Institute. Data were 72-base paired-end reads generated from Illumina GA IIx machines. Each sample was run on a single lane of a flowcell. DNA enrichment was done using the Agilent SureSelect Human All Exon kit v1, which was designed to target 165,637 genomic regions (37.8 million bases; 71.6% inside exons; average length 228 bp).

We shifted the Illumina base quality scores (Phred+64) to the Sanger scale (Phred+33) [6] and performed an initial alignment to the National Center for Biotechnology Information human reference genome HG19 using the program Burrows-Wheeler Aligner (BWA) [7]. We then marked duplicates with Picard and carried out regional realignment and quality score recalibration using the Genome Analysis Toolkit (GATK) [1]. For variant calling, we only used reads with a mapping quality score (MAPQ) ≥20 (i.e., ≤1% probability of being wrong) and bases with base quality score (BQ) ≥20. We used GATK's Unified Genotyper to call SNPs simultaneously on all samples.

The same 22 samples were genotyped using the Affymetrix Genome-Wide Human SNP Array 6.0 which features 1.8 million genetic markers, including more than 906,600 SNPs. Approximately one-third of the SNPs on the Affymetrix Genome-Wide Human SNP Array 6.0 reside in the exome regions covered by the Agilent SureSelect Human All Exon kit v2.

Two additional independent datasets were used to validate our findings. The first additional data set contains six samples, randomly selected from the 1000 Genomes Project [8], that were sequenced on the Illumina GAII with capturing performed with an array based method. The second additional dataset contains six samples sequenced on the Illumina HiSeq 2000 sequencer, with capturing performed with the Illumina TruSeq capture kit. The variety of capture methods and sequencers used to collect those data provided more robustness for our study.

### Strand bias scores

Sequencing data at a single position in the genome can be represented by a 2 by 2 table _*c*_^*a*^ _*d*_^*b*^ where a, c represent the forward and reverse strands allele counts of the major allele, and b, d represent the forward and reverse strands’ allele counts for the minor allele. Occasionally, reads can align to a third allele at a given genomic position. Such third alleles are likely due to sequencing or alignment errors. Thus we discarded the third allele from the analysis in the rare cases where it was observed. Several strand bias examples from real data can be viewed in Table 1. Based on the 2 by 2 table, we can calculate three scores to measure strand bias:

1. SB: SB is defined as $|\frac{b}{a+b}-\frac{d}{c+d}|/\left(\frac{b+d}{a+b+c+d}\right)$. The calculation of SB has been used previously in a mitochondria heteroplasmy study [9]

2. GATK-SB: GATK-SB is the strand bias score calculated by GATK [1], and it is defined as Max $\left[\left(\frac{b}{a+b}*\frac{c}{c+d}\right)/\left(\frac{a+c}{a+b+c+d}\right),\left(\frac{d}{c+d}*\frac{a}{a+b}\right)/\left(\frac{a+c}{a+b+c+d}\right)\right]$

3. Fisher Score: The Fisher Score is derived from Fisher p-value and is calculated in the standard way using the 2 by 2 table. To ensure directional consistency with the SB and GATK-SB scores, the Fisher Score is defined as 1 minus the p-value.

Both SB and GATK-SB scores have ranges from 0 to infinity, while the Fisher score has a range from 0 to 1. For all 3 scores as we have defined them, lower values mean less strand bias and higher scores mean a more severe strand bias.

### Strand bias and genotype quality

Genotype consistency between sequencing calls and genotyping chip calls has been used as a quality control for sequencing data [3]. For example, GATK has a built-in tool that uses genotyping chip consistency as a SNP quality recalibration criterion. We performed a consistency analysis between the genotype inferred from the exome sequencing data and the genotype inferred from the SNP chip data. When inferring genotypes using sequencing data, all the genomic positions can be divided into two major categories: homozygous and heterozygous. Strand bias has no effect on the quality of homozygous genotype calls, because regardless of how severe the strand bias might be, it lacks sufficient influence to force the genotype caller to make a false heterozygous inference. Thus, in our analysis, we only considered heterozygous SNPs called by GATK's Unified Genotyper. The consistency is defined as the number of heterozygous SNPs with a consistent genotype between the exome sequencing data and the SNP chip data divided by all overlapped (with exome sequencing) heterozygous SNPs in the SNP chip data.

The SNPs present in both the Affymetrix 6.0 SNP Chip and the exome sequencing data are only a small percentage of the SNPs identified by exome sequencing. Thus, we computed other genotype quality control parameters such as the transition/transversion (Ti/Tv) ratio, and the number of novel non-synonymous SNPs. The Ti/Tv ratio is around 3.0 for SNPs inside exons and about 2.0 elsewhere [10]; it also differs between synonymous and non-synonymous SNPs [11]. Because the target regions of exome capture kits often cover more than just exons, the Ti/Tv ratio for SNPs inside these target regions is expected to lie between 2.0 and 3.0 with the value depending on the fraction of exons inside target regions. We also compared the Ti/Tv ratios between SNPs with low or no strand bias and SNPs with extreme strand bias in novel SNPs and the SNPs reported in dbSNP. Furthermore, the number of novel non-synonymous SNPs can also be a very good indicator of the false positive rate. A study [12] have shown that only 200–300 novel nonsynonymous SNPs should be identified per person by exome sequencing; a higher number would likely indicate a higher false-positive rate.

### Cause of strand bias

To identify the cause of strand bias, we want to initially determine if the strand bias occurs systematically across subjects. Thus, we examined the strand bias score consistency between samples. For the 22 breast cancer samples, there are total of 231 possible pairs. For each pair, we selected positions in the top 20 percent of strand bias scores from one subject in the pair, and computed the Pearson correlation coefficient using the strand bias scores at the selected positions between the two samples in the pair. By only selecting the positions with high strand bias scores in one subject in the pair, we can effectively capture the scenario where two subjects have significantly different strand bias scores at the same positions. Box plots were used to show the distribution of the correlations across the 231 pairs.

We also hypothesized that post analysis procedures may also contribute to the cause of strand bias. After initial alignment, several popular enrichment steps are often used to reduce the genotyping false-positive rate. Such steps include local realignment, base quality score recalibration, base alignment quality recalibration (BAQ), and removal of duplicate reads. Based on the popularity of these steps, we examined the strand bias score using four different processing pipelines: (1) initial alignment (without performing any enrichment steps, denoted as initial alignment); (2) realignment, recalibration and removal of duplicates (denoted as realignment); (3) base alignment quality recalibration (denoted as BAQ); (4) local realignment plus BAQ (denoted as realignBAQ). We computed the Pearson's correlation coefficients, and plotted scatter plots of strand bias scores between each processing pipeline. Finally, we realigned our data using a second aligner Bowtie, and compared the strand bias scores computed from alignment bam files between Bowtie and BWA.

## Results

### SNP Chip genotyping quality

The 22 breast cancer patient samples sequenced with the Agilent SureSelect capture kit were taken from 2776 patients who were genotyped using the Affymetrix SNP 6.0 array in a genome-wide association study; detailed genotyping methods and stringent QC criteria were described in Zheng et al. [4]. The original scan included three quality control samples in each 96-well plate, and the SNP calls showed a very high concordance rate (mean 99.9%; median 100%) for the quality control samples. In addition, 742 SNPs were genotyped using alternative genotyping platforms for a subset of subjects; these SNPs also had a high concordance rate with genotypes obtained from the SNP chip (mean 99.1%; median 99.8%). The SNP chip call rate for the 22 samples investigated here ranged from 97.83% to 97.84%.

### Sequencing data quality

Our sequencing data has high quality. Table S1 contains detailed summaries of the samples studied. For the 22 samples sequenced with the Agilent SureSelect capture kit, we obtained an average of 68.9 (range 44.6-78.2) million reads per subject, with 45x median depth for the SureSelect target regions. On average, 91.4% (88.4-93.8%) of the reads were aligned to the human reference genome, and 86.2% (82.1-89.3%) had an insert size ≤ 500. The six samples sequenced with the Illumina TrueSeq capture kit had an average of 93.8 (range 91.3-98.0) million reads and achieved 48x median depth for the TrueSeq target regions. On average, 95.1% (94.9-95.6%) of the reads had insert size ≤ 500. The six samples sequenced by the 1000 Genomes Project had an average of 67.9 (range 47.5-83.7) million reads and achieved 59x median depth for their target regions. On average, 89.7% (72.1-99.1%) of the reads had insert size ≤ 500.

### Strand bias and genotyping quality

**Figure 1:**
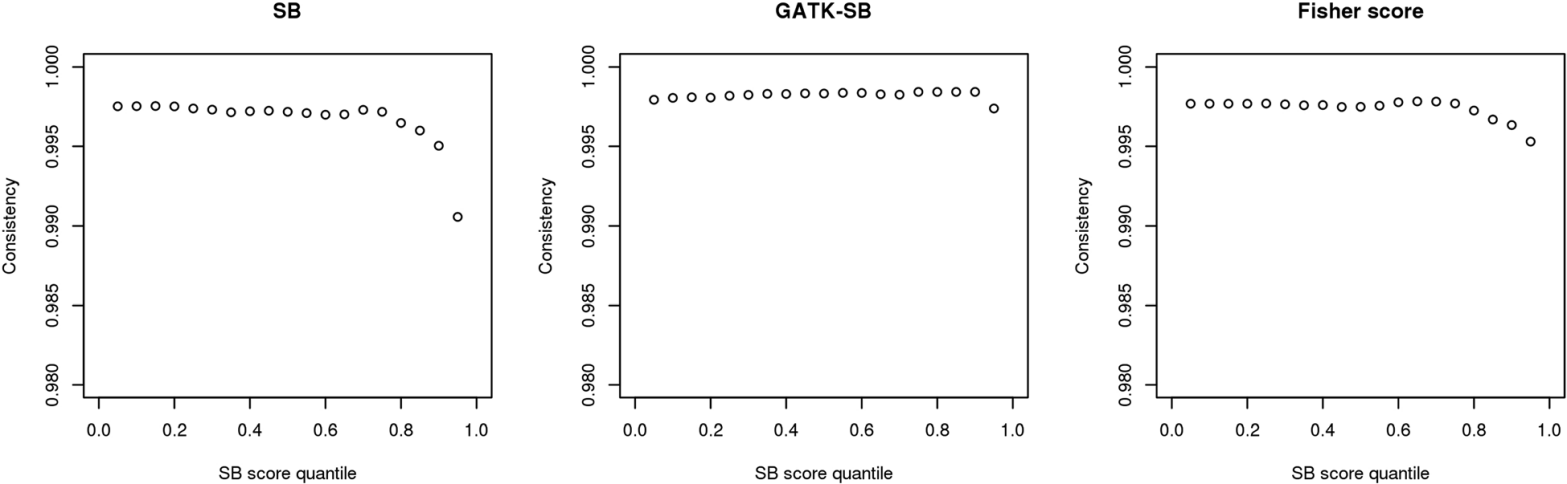
Consistency rates with SNP chip data using 3 strand bias scores and 4 processing pipelines.

To evaluate the effect of strand bias on genotype quality, we plotted the strand bias scores quantile (5% each) against the genotype consistency between genotypes inferred from exome sequencing data and SNP chip data (Figure 1). The strand bias scores are more accurate if high numbers of reads are observed on both strands; thus, we used a forward depth ≥ 10 and a reverse depth ≥ 10 as a filter. We observed that, for both the SB and Fisher scores, a minor drop in the heterozygous genotype consistency rate (< 1%) was detected as strand bias scores increased from the 80 percentile to the 100 percentile. We hypothesized that Affymetrix’s SNP selection criteria selected SNPs that are more easily sequenced by exome sequencing technology. Thus, the overlapping subset of SNPs has better quality than the rest of the SNPs and the consistency rate for this subset of SNPs might not represent the overall quality of SNPs identified by exome sequencing. To test this hypothesis, we compared the SNPs on the Affymetrix 6.0 chip with the rest of the SNPs identified by the exome sequencing, grouped by minor allele frequency (MAF) (Table 2). The dbSNP and exome sequencing overlap subset had a Ti/Tv ratio of 2.26, which is higher than the average Ti/Tv ratio of 2.08 for all sequenced SNPs. This is consistent with our previous finding [3] that showed a higher quality for dbSNP SNPs than for novel SNPs. This can be easily explained by the higher amount of false-positives in novel SNPs compared to the previously reported SNPs. The most interesting observation was that the overlapping SNPs between the exome sequencing and the genotyping chip (also a subset of dbSNP SNPs) had a higher Ti/Tv ratio than the rest of the dbSNP SNPs (2.78 vs 2.21). This evidence supports our hypothesis that the Affymetrix 6.0 genotyping chip’s SNPs are more easily sequenced than the full set of SNPs reported by the exome sequencing.

**Table 2:** **SNP quality by MAF and subset**^1^

	**MAF 0–0.1**	**MAF 0.1-0.2**	**MAF 0.2-0.3**	**MAF 0.3-0.4**	**MAF0.4-0.5**	**Overall**
All Seq SNPs	70825/2.35	8454/1.93	26074/1.94	19092/1.96	30532/1.85	174977/2.08
Overlapped SNPs	4123/2.99	2781/2.8	2799/2.73	2160/2.53	2504/2.69	14367/2.78
Seq SNPs - Chip SNPs	66702/2.31	25673/1.86	23275/1.87	16932/1.9	28028/1.79	160610/2.03
dbSNP SNPs in Seq	53520/2.43	23458/2.21	21798/2.23	16359/2.2	26152/2.05	141287/2.26
dbSNP SNPs not on Chip	49397/2.39	20677/2.15	18999/2.17	14199/2.16	23638/2.00	126920/2.21

^1^. Each cell is represented in the format of number of SNPs/TiTv ratio.

**Figure 2:**
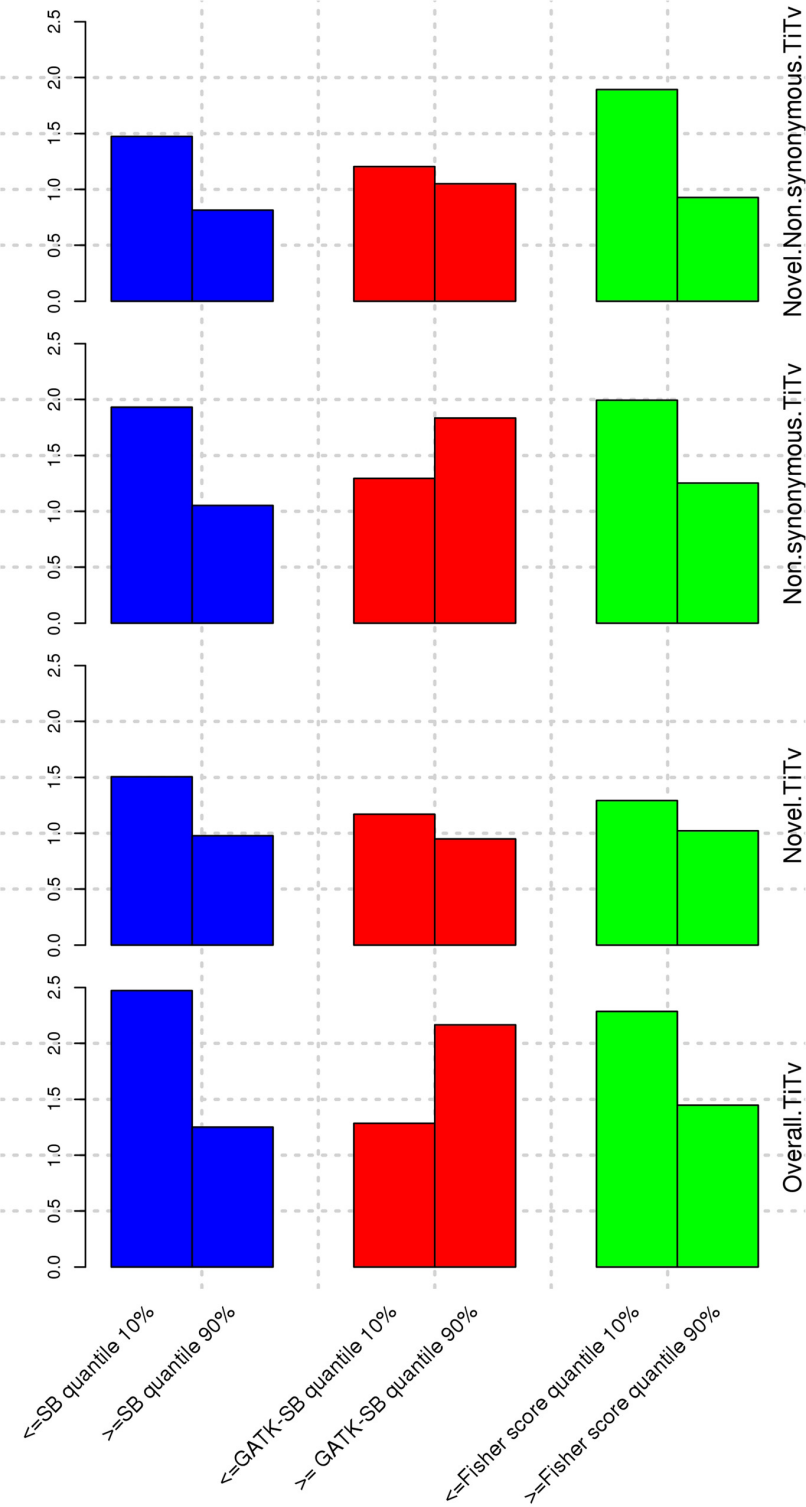
Genotyping quality control through Ti/Tv ratio.

Furthermore, we performed quality control tests on SNPs with low or no strand bias (bottom 10%) and SNPs with high bias (top 10%) (Figure 2). For the SB and Fisher scores, we observed an overall better quality for SNPs with low or no strand bias than for SNPs with extreme bias. For example, using the SB score, we identified, on average, a Ti/Tv ratio of 2.47 for SNPs with low or no strand bias, while the Ti/Tv ratio for SNPs with extreme bias was 1.25. For novel SNPs, the Ti/Tv ratio was 1.51 and 0.98 for SNPs with low and high strand bias, respectively. However, using the SB-GATK score, we observed some misleading results. For example, using the SB-GATK score, we identified an average Ti/Tv ratio of 1.3 for SNPs with low or no strand bias, while the average Ti/Tv ratio for SNPs with extreme bias was 2.2. For novel SNPs, the average Ti/Tv ratio was 1.2 and 1.1 for SNPs with low and high strand bias, respectively, according to GATK-SB score. For the number of non-synonymous SNPs, similar contradictory results between the SB, the Fisher scores and the GATK-SB score were observed. SNPs with low or no strand bias contained a reasonably low number of novel non-synonymous SNPs, and SNPs with extreme bias contained an unreasonably higher number of novel non-synonymous SNPs according to the SB and Fisher scores while the GATK-SB score showed the opposite results. Upon further investigation, we found that for certain situations, GATK-SB can produce a different score from the SB and Fisher scores. Three examples are given in Table 3. In all three examples, the non-reference allele has low counts on both strands. In case where b << a and d << c, the definition of GATK-SB simplifies to $\max\left[\frac{b}{a},\frac{d}{c}\right]$ and both choices are small. In contrast, the SB measure goes to infinity as b << a and d << c, as long as $\frac{b}{a}\neq \frac{d}{c}$. The SB and Fisher scores indicated quite strong strand bias at these three given genome positions while GATK-SB indicated a low strand bias for those positions. Furthermore, GATK’s Unified Genotyper made different genotype calls from the SNP chip data for all three genome positions. It is very likely that GATK’s Unified Genotyper made the wrong genotype calls on these positions. Thus we see an artifact where the SNPs with higher strand bias actually have higher Ti/Tv ratios than SNPs with low or no strand bias for the GATK-SB score. This result shows that the GATK-SB score is capturing a different group of SNPs than the SB and Fisher scores in the situation described in Table 2. The complete results of genotype quality control can be found in Table S2.

**Table 3:** SB and GATK-SB difference

**Chr**	**Pos**	**a**^**1**^	**b**^**2**^	**c**^**3**^	**d**^**4**^	**SB**	**GATK-SB**	**Fisher**	**Genotype GATK**	**Genotype GWAS**
7	43917013	11	2	20	0	2.54	0.16	0.85	AG	AA
14	95923670	16	2	10	0	1.56	0.12	0.48	CT	TT
19	57088850	8	2	16	0	2.6	0.21	0.86	AC	AA

^1^. Forward strand reference allele.

^2^. Forward strand non reference allele.

^3^. Reverse strand reference allele.

^4^. Reverse strand non reference allele.

### Causes of strand bias

**Figure 3:**
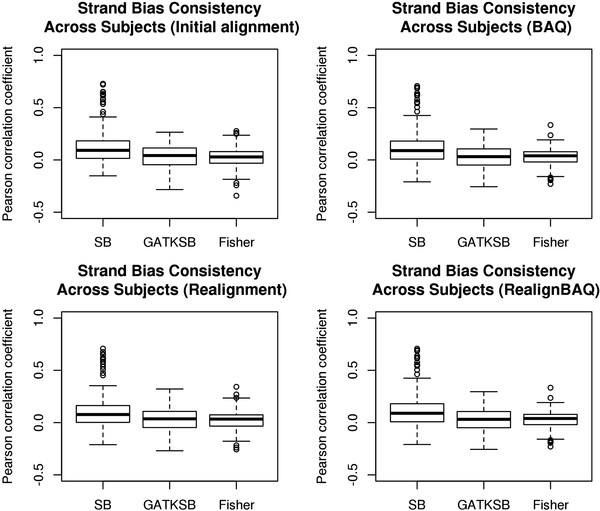
Strand bias consistency across subjects.

To study strand bias in greater detail we explored its repeatability across samples. For the 22 breast cancer samples, we randomly paired 2 samples and computed the Pearson's correlation coefficient between their strand bias scores. We plotted box plots of Pearson's correlation coefficients of all three strand bias scores for all possible 231 pairs of subjects (Figure 3). Unlike unbalanced strands that we have previously shown to occur consistently across subjects due to capture artifacts [3], no consistent pattern was detectable for strand bias. The median correlation coefficients of all three strand bias scores were near zero, suggesting that strand bias does not occur consistently at the same genomic sites across subjects.

**Figure 4:**
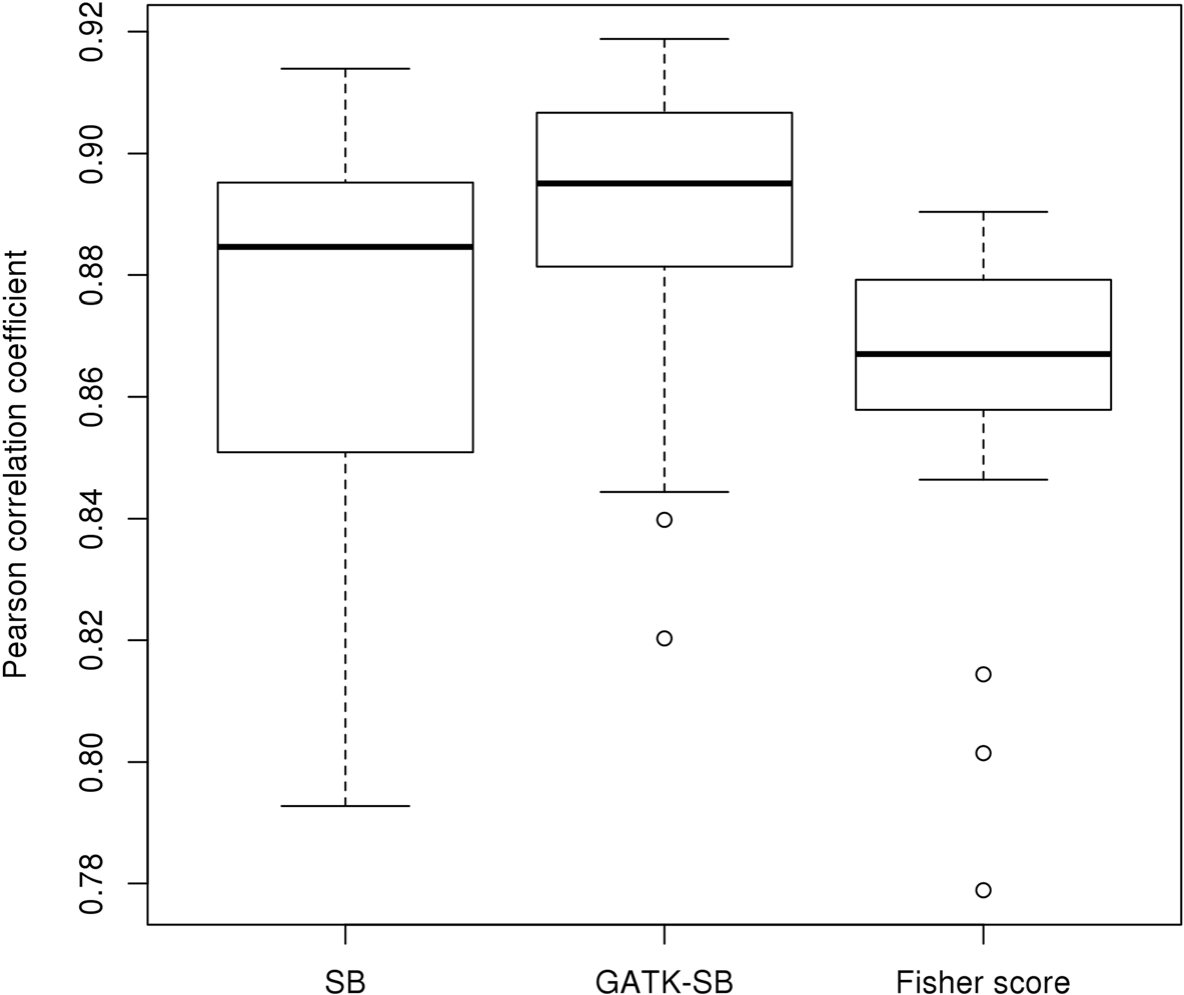
Strand bias correlations between Bowtie and BWA.

To determine the role of the alignment method in generating strand bias, we aligned our samples using an additional aligner: Bowtie [13]. Then we computed the Pearson's correlation coefficients of all three allele frequency scores between the BWA and Bowtie alignments (Figure 4). High correlations (r >0.85) were observed for all three strand bias scores between BWA and Bowtie alignments. This result suggests that strand bias is likely not an artifact of alignment, since high levels of strand bias at the same genomic sites were reproduced using these different alignment methods. However, we cannot completely rule out the possibility that both aligners produced similar artifacts that caused similar strand bias.

**Figure 5:**
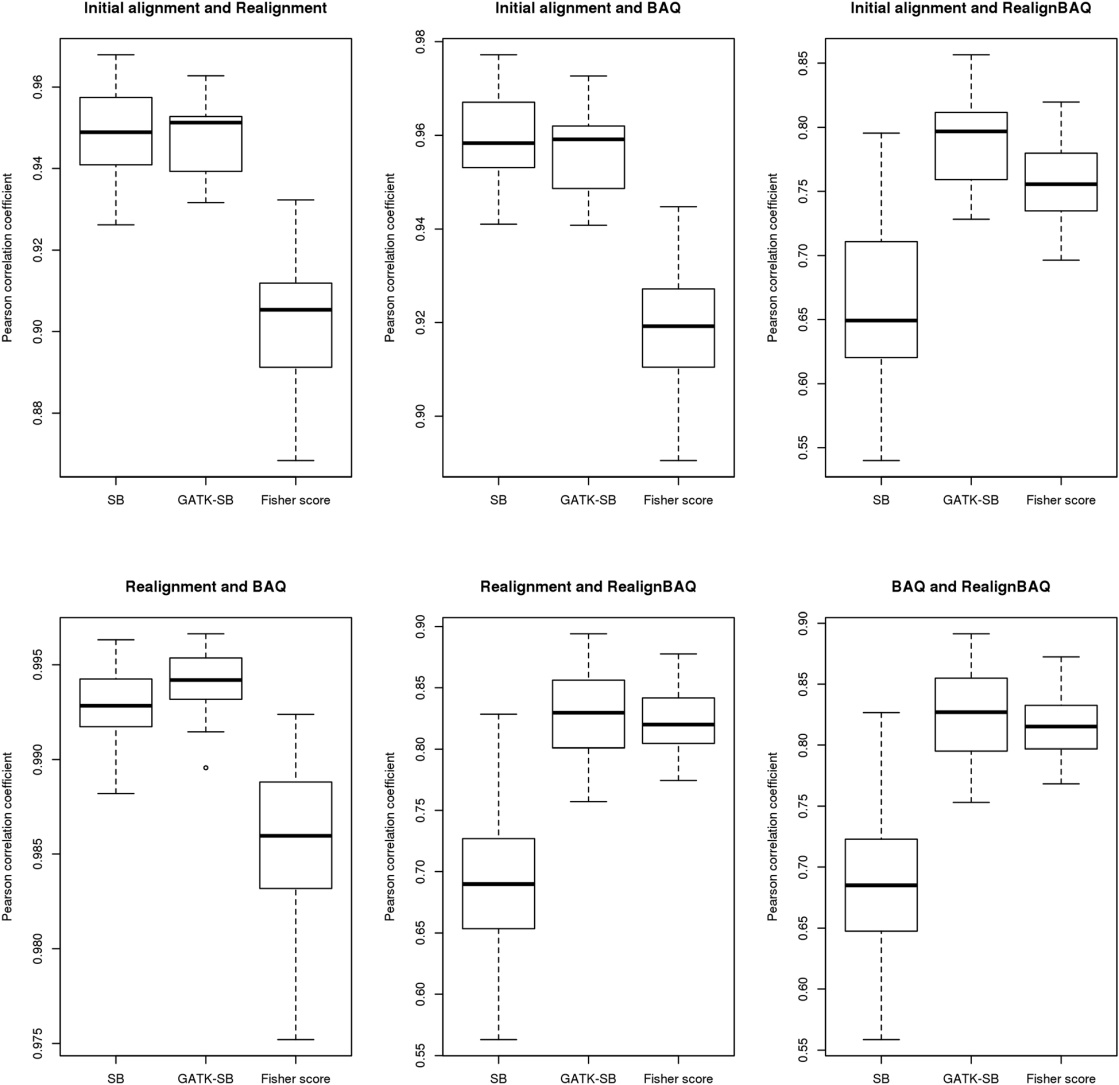
Correlation of strand bias scores between different processing pipelines.

**Figure 6:**
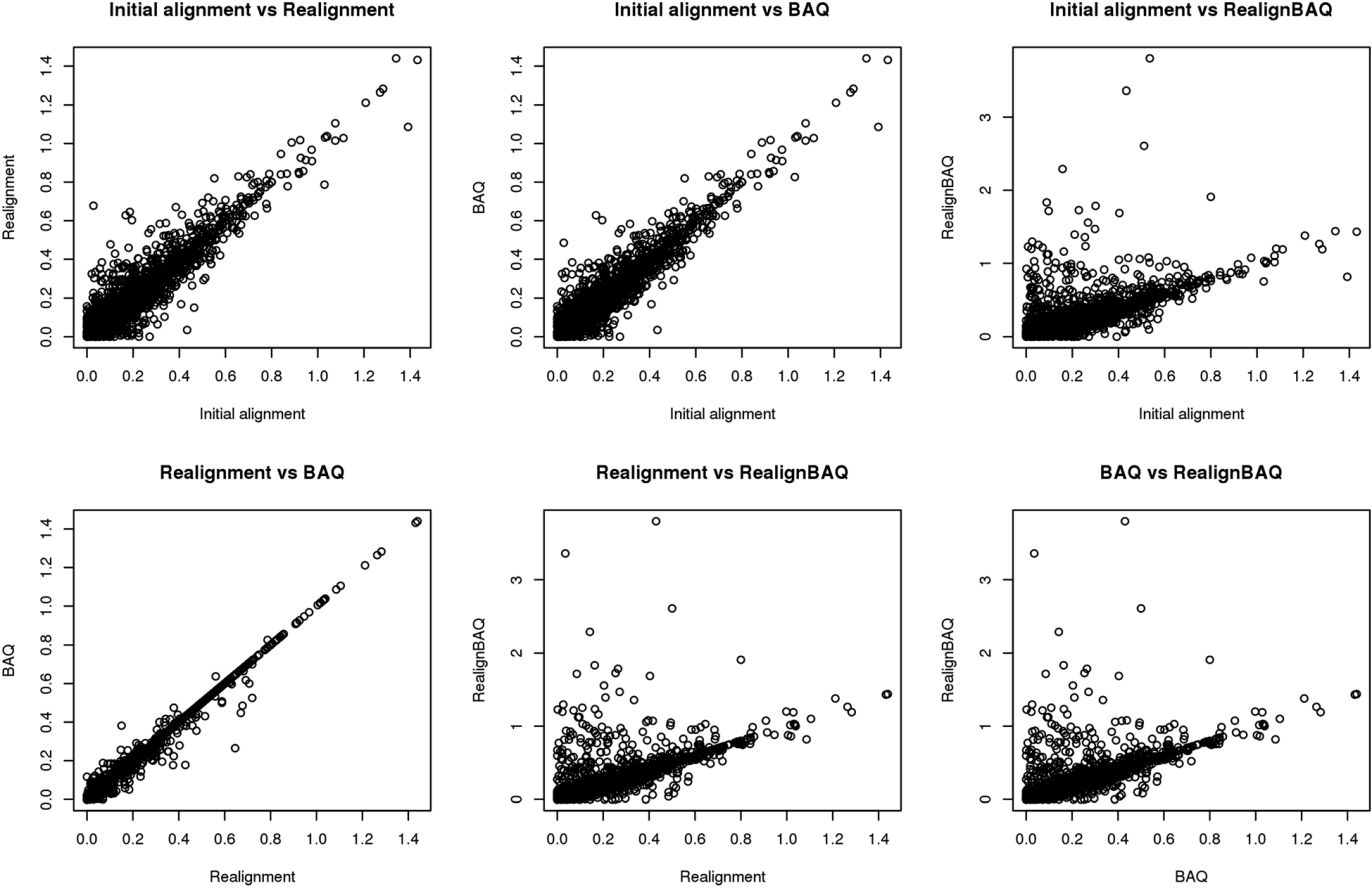
Scatter plot of strand bias scores between different processing pipelines.

We calculated Pearson's correlation coefficients for all three strand bias scores among the four processing pipelines that we have described in the Methods section. High correlations (median r > 0.90) were observed between the strand bias scores computed from the different pipelines, except for the RealignBAQ pipeline (median r < 0.70) (Figure 5). These results suggest that the post analysis procedures such as local realignment, recalibration, and BAQ did not contribute to the causes of strand bias. However, the pipeline using local realignment plus BAQ had relatively low strand bias correlation with other processing pipelines. From the scatter plot (Figure 6), we can also see that realignBAQ introduced more SNPs with higher strand bias. The phenomenon observed for realignBAQ is primarily due to the redundancy between local realignment and BAQ. Both the local realignment and BAQ procedures are designed to lower the SNP false-positive rate, one by adjusting the alignment and the other by adjusting the base quality score around indel regions. Applying both in the same pipeline (as we did in the RealignBAQ pipeline) produced an adverse effect causing larger strand biases.

All analyses described were repeated using two additional independent datasets. Similar findings were observed without exception. The results for the additional datasets can be viewed in the supplementary material.

## Discussion

Due to its vast popularity and easily accessible data, we focused our study on exome sequencing data from the Illumina sequencing platform. We did not evaluate strand bias on data generated by other sequencing platforms, such as 454 Life Science and Applied Biosystems. We speculate that the same phenomenon exists for the Applied Biosystems sequencing platform, because its technology can generate a similar depth of data compared to Illumina’s platform. However, it would be hard to observe or study strand bias on the 454 Life Science’s sequencing platform, due to its limited depth.

An interesting finding from this study is that sequencing data and genotyping chip consistency might not represent the whole picture of sequencing data quality, as we previously thought. The overlapping subset of SNPs is small compared to all SNPs identified through exome sequencing. Also, all SNPs on the standard genotyping chips are also in dbSNP and the overlapping portion is not a random sample from all SNPs identified by exome sequencing. The exact SNP selection criteria for the Affymetrix 6.0 SNP chip are unknown to us. We speculate that criteria such as GC content, proximity to other SNPs and the ease of enzyme ligation should be considered; these factors also impact the sequencing quality. We observed a better quality for genotyping chip SNPs in sequencing, raising concerns about the effectiveness of using genotyping chip consistency rate as a quality control for SNPs identified by sequencing. We believe that the genotyping chip consistency can still be used as a quality control; however, the use of other quality control parameters, such as the Ti/Tv ratio and the number of novel non-synonymous SNPs in conjunction, is essential for obtaining an accurate description of the SNPs identified by sequencing.

## Conclusions

We found that strand bias does not consistently occur at the same genomic sites across different samples. By comparing strand bias using different post-analysis pipelines, we found some evidence to support the hypothesis that post-analysis procedures can cause strand bias, especially for the processing pipeline that applies both local realignment and BAQ. Such processing pipelines can introduce more SNPs with higher strand bias, which in turn results in more false-positive SNPs. Use of local realignment and BAQ in the same processing pipeline should be avoided. The correlation of allele frequency scores between BWA and Bowtie were very high, indicating that strand bias is not likely due (although not completely ruled out) to the artifacts of alignment, but is more likely an artifact or due to errors from the library preparation or sequencing. A portion of the strand bias can also be caused by sampling variation during the sequencing.

For the three strand bias scores we have studied, SB, GATK-SB, and Fisher scores, we evaluated their effectiveness in capturing true false-positive SNPs. Based on our results, the Fisher and SB scores can capture true false-positive SNPs better than the GATK-SB score. By comparing exome sequencing data with SNP chip data, the SB and Fisher scores indicated slight drops in heterozygous consistency when strand bias scores were over the 80th percentile. However, the magnitude of the consistency rate drop is minor. From the other genotype quality control parameters, such as Ti/Tv ratio and number of novel non-synonymous SNPs, we observed an overall better quality for SNPs with low or no strand bias than for SNPs with extreme strand bias. Based on our findings, strand bias can negatively affect the genotyping quality of sequencing data. We recommend caution when applying strand bias as a filter. Only SNPs with extreme strand bias should be regarded as false-positive candidates. We considered SNPs with strand bias score in the top 10% as extreme. VarScan [14], a variant calling tool also uses the top 10% rather than a fixed numerical score as a strand bias filter. Indiscriminant use of strand bias as a filter will result in a large loss of true positive SNPs.

## Competing interest

The authors declare that they have no competing interest.

## Authors' contributions

YG, JL and C-I performed the analysis. JL processed the original data. DS and YS provided significant scientific input. YG wrote the manuscript. All authors read and approved the final manuscript.
